# Limited impacts of dietary Protandim Nrf2 Synergizer on antioxidant and inflammatory status of mature, sedentary horses

**DOI:** 10.1093/jas/skaf433

**Published:** 2025-12-15

**Authors:** Pier L Semanchik, Lauren T Wesolowski, Jessica L Artman, R Lee Seward, Christina Beer, Elisa D Barnes, Sarah H White-Springer

**Affiliations:** Department of Animal Science, Texas A&M University and Texas A&M AgriLife Research, College Station, TX 77843; Department of Animal Science, Texas A&M University and Texas A&M AgriLife Research, College Station, TX 77843; Department of Animal Science, Texas A&M University and Texas A&M AgriLife Research, College Station, TX 77843; LifeVantage Corporation, Lehi, UT 84043; LifeVantage Corporation, Lehi, UT 84043; LifeVantage Corporation, Lehi, UT 84043; Department of Animal Science, Texas A&M University and Texas A&M AgriLife Research, College Station, TX 77843; Department of Kinesiology and Sport Management, Texas A&M University, College Station, TX 77843

**Keywords:** antioxidant, horse, inflammation, Protandim

## Abstract

Reactive oxygen species are normal by-products of cellular metabolism but may have detrimental effects on cellular matrices and excite inflammatory pathways when overproduced. To test the hypothesis that supplementation of an herbal extract combination would: 1) improve antioxidant status; 2) increase anti-inflammatory cytokines; and 3) decrease pro-inflammatory cytokines, 40 mature, sedentary stock-type horses (32 mares, 8 geldings, mean±SD; 15.7 ± 4.9 yr, 519 ± 46 kg) were stratified by age, sex, and body weight and randomly assigned to one of four dietary treatment groups for 56 d: 1) 0 mg (CON); 2) 675 mg (Pro1); 3) 2,025 mg (Pro3); or 4) 4,050 mg (Pro6) Protandim Nrf2 Synergizer (LifeVantage Corporation) per day (*n* = 10/group). Horses were group housed and received a basal diet of mixed warm-season grass pasture and hay ad libitum and a custom-formulated concentrate grain. Blood collected prior to the morning feeding on day 0, 28, and 56 was analyzed for hydrogen peroxide (H_2_O_2_) production and concentration, superoxide dismutase (SOD), glutathione peroxidase (GPx), and catalase (CAT) activities, concentrations of malondialdehyde (MDA), cytokines [interleukin (IL)-4, IL -6, IL -8, IL -10 and tumor necrosis factor α], and caffeine, and mRNA expression of *IL -1β*, *Nrf2*, and *HMOX1*. Activities of GPx and SOD were also quantified in gluteus medius samples collected at day 0 and 56. Data were analyzed using linear models in SAS v9.4; sex, time, treatment, and time×treatment were fixed effects and time was a repeated effect with horse(treatment) as the subject. Plasma caffeine concentrations increased from day 0 to 56 in supplemented horses (*P *≤ 0.05) in a dose-dependent fashion but did not change in CON horses, resulting in Pro6 horses having the greatest concentration of caffeine at day 56, followed by Pro3, Pro1, then CON horses. No other measure was impacted by treatment though whole blood H_2_O_2_ production, SOD activity, and *IL -1β* mRNA, and plasma IL -8 and MDA concentrations decreased by day 28 (*P * ≤0.006), whole blood *Nrf2* mRNA and IL -10 concentrations decreased by day 56 (*P*≤0.04), and skeletal muscle GPx activity increased by day 56 (*P = *0.05) in all horses. Dietary supplementation of up to 4,050 mg/d Protandim Nrf2 Synergizer did not impact antioxidant status or plasma cytokines in mature, sedentary horses. Effects of supplementation on these variables should be investigated in horses subjected to elevated oxidative and/or inflammatory insult, such as during exercise or aging.

## Graphical abstract

**Graphical Abstract:**
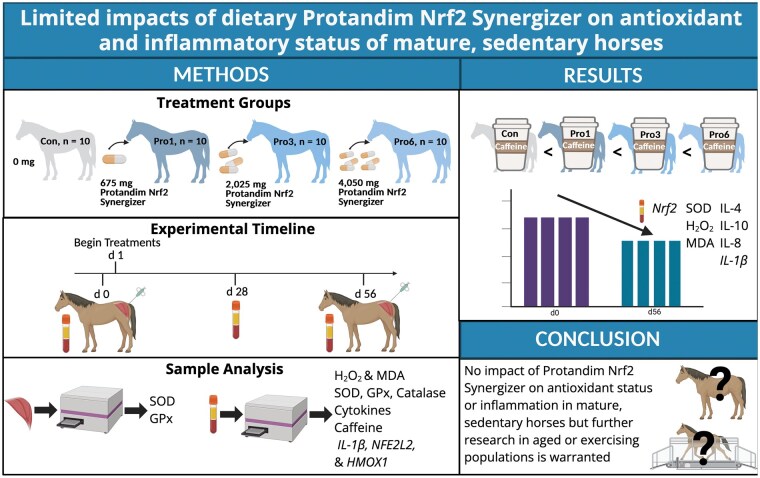

## Teaser

Protandim supplementation up to 4,050 mg per day did not impact antioxidant defense systems or inflammatory markers in mature, sedentary horses. Further evaluation using a greater dosage, an exercising equine athlete model, or aged horses should be considered.

## Introduction

Reactive oxygen species (ROS) are normal by-products of cellular metabolism that act as essential signaling molecules, inducing adaptation (Sies and Jones, 2020). However, when ROS production surpasses the capacity of antioxidant defenses, oxidative stress ensues, leading to deleterious interactions with cellular components. The resulting accumulation of ROS can cause oxidative damage to proteins, lipids, DNA, and cellular membranes (Ďuračková, 2010), disrupting normal cellular function. Reactive oxygen species are also key mediators of inflammation. When produced in excess, ROS activate transcription factors such as nuclear factor-kappa B (NF-κB), leading to the upregulation of pro-inflammatory cytokines and chemokines (Kim et al., 2017). Reactive oxygen species also contribute to the activation of the NOD-like receptor family pyrin domain containing 3 (NLRP3) inflammasome, which facilitates the maturation and secretion of interleukin-1β (IL-1β) and IL-18, further amplifying inflammation (Dominic et al., 2022). Additionally, ROS-induced oxidative modifications of cellular macromolecules generate damage-associated molecular patterns (DAMPs), which are recognized by pattern recognition receptors, such as Toll-like receptors (TLRs), thereby perpetuating sterile inflammatory responses (Koenig and Buskiewicz-Koenig, 2022). To combat the onset of oxidative stress and ROS-associated inflammation, antioxidants work to neutralize free radicals and maintain the balance of oxidants to antioxidants.

Dietary antioxidants are found in various forms and can be supplemented to mitigate ROS accumulation. For example, supplementation with vitamin E and/or selenium has been associated with decreased ROS and improved mitochondrial capacities in young, exercising horses (White et al., 2016; Owen et al., 2022). Similarly, providing a mixed antioxidant supplement to exercising 2-yr-old Thoroughbreds reduced baseline and attenuated post-exercise increases in mRNA expression of pro-inflammatory cytokines, *TNFα* and *IL-1β* (Horohov et al., 2012). Although limited research has examined antioxidant supplementation in aged horses, chronic low-grade inflammation, termed “inflammaging,” is well documented in this population (Adams et al., 2008; Adams et al., 2009; DeNotta and McFarlane, 2023). Evidence suggests that antioxidant supplementation to aged horses may reduce inflammatory markers following transportation stress (Miller et al., 2021) and help prevent seasonal immune disruptions (Kędzierski et al., 2020), highlighting a potential link between antioxidant strategies and inflammation management.

There are numerous sources of antioxidants, both natural and synthetic. However, the equine industry is faced with breed and competition regulations that restrict the types of antioxidants that can be supplemented. To combat this, herbal antioxidant supplements are becoming a field of interest. For example, in humans, herbal blends containing turmeric or green tea have been shown to decrease lipid peroxidation and increase antioxidant capacity (Jakubczyk et al., 2020; Osada et al., 2001). Strategies to mitigate oxidative stress have also shifted from the use of isolated antioxidant supplements toward approaches that enhance the body’s endogenous antioxidant defense systems. One such approach is the stimulation of nuclear factor erythroid-derived 2-like 2, or Nrf2, which is known as the “master regulator” of the antioxidant system and is also known to induce an anti-inflammatory cellular profile (Hybertson et al., 2011; Vomund et al., 2017). In humans, Nrf2 and a primary gene it upregulates, heme oxygenase-1 (HMOX1), are postulated to be critical in the prevention of age-related decline (O’Rourke et al., 2024). As such, supplements containing ingredients known to stimulate Nrf2 have gained traction. One such supplement is LifeVantage Protandim Nrf2 Synergizer, a proprietary blend of milk thistle, bacopa, ashwagandha, green tea, and turmeric. Protandim has been shown to decrease oxidative stress in a mouse model of muscular dystrophy (Qureshi et al., 2010), improve skeletal muscle contractile protein production in aged humans (Konopka et al., 2017), and decrease oxidative stress while maintaining oxidant-induced exercise adaptations in rats (Bruns et al., 2018).

Given the reported benefits of Protandim supplementation in other species across various physiological conditions, we aimed to investigate the impacts of LifeVantage Protandim Nrf2 Synergizer supplementation on the horse’s antioxidant and inflammatory systems. We hypothesized that horses receiving LifeVantage Protandim Nrf2 Synergizer for 56 d would have lower markers of oxidative and inflammatory stress compared to horses not receiving supplementation. Further, we expected an inverse linear relationship of Protandim dose with oxidative and inflammatory stress markers.

## Materials and Methods

This study was reviewed and approved by the Institutional Animal Care and Use Committee at Texas A&M University (2019-0331).

### Horses

Forty mature stock-type horses (*n* = 8 geldings, *n* = 32 mares) aged 7 to 23 yr (mean ± SD; 15.7 ± 4.9 yr) with an average body weight (BW) of 519 ± 46 kg were sourced from the Texas A&M University Veterinary Medical Park (College Station, TX) where they remained during a 28-d background period and 56-d trial. All horses were group housed in four separate pastures (pasture 1: *n* = 11 mares; pasture 2: *n* = 10 mares; pasture 3: *n* = 8 geldings; pasture 4: *n* = 11 mares) approximately 2.4 hectares in size and were individually fed a concentrate grain twice daily in outdoor panel stalls. Pastures each shared a fenceline so similar fresh forage was available in all pastures. Horses were void of a structured exercise regime.

### Dietary treatments

Prior to the onset of supplementation, horses were maintained on a basal diet of a custom-formulated concentrate grain (Purina Mills, LLC, St Louis, MO) fed individually at 0.5% BW/d (dry matter basis; DM) split evenly between two meals at 0630 and 1700 and ad libitum mixed warm-season grass pasture and round bales for 28 d. Prior to the initiation of the trial, grain samples were analyzed to confirm nutrient composition by Equi-Analytical (Ithaca, NY) for complete analysis and by Eurofins (DeSoto, TX) for vitamin E and selenium content using standard wet-chemistry analytical methods (Table 1). The basal diet was formulated to meet or slightly exceed requirements for a mature horse at maintenance (NRC, 2007) with vitamin E and selenium levels limited to just meeting requirements to minimize additive antioxidant effects. The basal diet did not contain the active ingredients under investigation (milk thistle, bacopa, ashwagandha, green tea, and turmeric). The concentrate was provided at levels to maintain a body condition score (BCS) of 5 to 6 on the Henneke scoring scale (Henneke et al., 1983). Horses were weighed using a livestock scale and body condition scored by three independent investigators once every two weeks to ensure maintenance of a 5 to 6 BCS; the grain concentrate was adjusted as necessary.

**Table 1.** Nutrient composition of the custom-formulated concentrate, round bales, and warm-season grass pasture offered to mature, sedentary horses.

Nutrient^1^	Concentrate^2^	Hay	Pasture
**DE, Mcal/kg**	2.71	1.8	2.06
**CP, %**	14.15	10.6	14.2
**Starch, %**	20.25	1.8	2.8
**Crude fat, %**	5.20	1.8	3.3
**Ca, %**	2.68	0.53	0.46
**P, %**	0.72	0.21	0.35
**Mg, %**	0.32	0.16	0.2
**K, %**	1.13	1.95	2.29
**Na, %**	0.35	0.043	0.039
**Cl, %**	0.82	0.64	0.69
**S, %**	0.36	0.53	0.4
**Fe, ppm**	858.50	153	286
**Zn, ppm**	368	31	62
**Cu, ppm**	92.50	9	8
**Mn, ppm**	288	40	125
**Mo, ppm**	1.75	1	1.5
**Co, ppm**	0.89	0.16	0.43
**Se, ppm**	0.56	<0.25	0.102
**α-tocopherol, mg/100g**	27.55	1.86	2.38
**Total tocopherols, mg/100 g**	31.55	3.11	2.98

1 Values presented on a 100% dry matter (DM) basis.

2 Concentrate = basal grain fed to all horses at 0.5% body weight (BW) in DM per day.

On day 14 relative to the onset of supplementation, all horses were weighed to balance treatment groups by BW. Horses were then stratified by age, sex, and BW and randomly assigned to one of four treatment groups for 56 d: 1) 0 mg LifeVantage Protandim Nrf2 Synergizer (LifeVantage Corporation, Lehi, UT; CON); 2) 675 mg Protandim/d (Pro1); 3) 2,025 mg Protandim/d (Pro3); or 4) 4,050 mg Protandim/d (Pro6; *n* = 10 per group). Pasture assignments were also balanced by dietary treatment group. The dose provided to Pro6 horses (8.1 mg/kg BW based on an average 500-kg horse) was designed to be similar to the recommended dose in humans (8.4 mg/kg BW). Starting at day 0, the supplement was top-dressed in pill form once daily on the morning grain feeding. If horses sorted and did not consume the pill, the pill was ground into a fine powder prior to being top-dressed on the concentrate grain. With this slight modification, all horses consumed their allotted treatment.

### Sample collection

Blood samples were collected via jugular venipuncture at day 0 (mean environmental temperature 35.3 ± 0.7°C), 28 (25 ± 4.1°C), and 56 (12.6 ± 3.1°C) prior to the morning feeding into tubes with 1) clot activator for collection of serum (BD Vacutainer^®^, Franklin Lakes, NJ); 2) sodium heparin for collection of whole blood and plasma (BD Vacutainer^®^); and 3) Tempus Blood RNA Tubes (Applied Biosystems, Waltham, MA). Serum samples were allowed to clot at room temperature and sodium heparin and Tempus blood tubes were immediately placed on ice. Blood was processed within 2 h of collection. Whole blood was collected from the sodium heparin tubes and the remaining heparinized blood was centrifuged at 1500×*g* for 10 min at 4°C and serum tubes were centrifuged at 1500×*g* for 10 min at 25°C after clotting. Serum samples were immediately analyzed, Tempus tubes were stored at −20°C, and all other sample types were divided into small aliquots and stored at −80°C for later testing.

Muscle tissue was collected from the gluteus medius muscle at day 0 and 56 of supplementation, alternating the left and right sides of the horse. The gluteus medius was isolated by tracing a line from the tailhead to the tuber coxae and collecting samples at one-third of that distance from the cranial end (Bechtel and Kline, 1987). Prior to tissue collection, horses were sedated by intravenous administration of detomidine hydrochloride (Dormosedan; Zoetis, Parsippany-Troy Hills, NJ) at dosages recommended by the manufacturer and the collection site was prepared as previously described (White et al., 2016). Briefly, the site was surgically clipped and cleaned with povidone-iodine scrub, then rinsed with 70% ethyl alcohol. After the site was cleaned, a 12-gauge needle was used to puncture the skin, and the muscle tissue was collected using a minimally invasive 14-gauge × 9 cm tissue collection needle (SuperCore^TM^ Semi-Automatic Biopsy Instrument, Argon Medical Devices, Plano, TX) inserted to a depth of 5 cm with an insertion angle of 90°. The muscle samples were snap-frozen in liquid nitrogen and stored at −80°C for later analysis. After muscle collection, the site was cleaned with 70% ethyl alcohol and sealed using aluminum bandage. Horses were monitored for 1 wk post-collection for adverse reactions, and none were noted.

### Caffeine concentrations

Sodium heparin plasma aliquots at day 0 and 56 were sent to the Texas A&M Veterinary Medical Diagnostic Laboratory (College Station, Texas) for caffeine analyses using liquid chromatography-tandem mass spectrometry.

### Markers of antioxidant status

Whole blood aliquots were analyzed for superoxide dismutase (SOD), glutathione peroxidase (GPx), and catalase (CAT) activities on a microplate reader (BioTek Synergy H1, Agilent Technologies, Santa Clara, CA) via spectrophotometry using commercially available kits (Cayman Chemical Company, Ann Arbor, MI); skeletal muscle was also analyzed for SOD and GPx activities. Skeletal muscle was first cryopulverized (Spectrum Bessman Tissue Pulverizer; Spectrum Laboratories, Inc.) into a fine powder then sonicated (F60 Sonic Dismembrator, Fisher Scientific) in extraction buffer (1 mM EGTA, 210 mM mannitol, 70 mM sucrose, pH 7.2) 3 × 3 s on ice. Tissue (mg):buffer (µL) ratios were 1:80 and 1:40 for SOD and GPx, respectively. Homogenates were then centrifuged for 15 min at 10,000×*g* at 4°C and supernatants harvested. A 1:150 dilution of whole blood with kit diluents was used for analysis of SOD and GPx at an absorbance of 450 and 340 nm, respectively. One reported unit of SOD is the enzymatic activity required to cause a 50% dismutation of the xanthine oxidase generated radicals detected by tetrazolium salt. GPx activity was determined indirectly through a coupled reaction with glutathione reductase. A 1:4,000 dilution with kit diluents was used for whole blood CAT analysis and was based on the reaction of CAT with methanol in the presence of optimal H_2_O_2_ concentrations at an absorbance of 540 nm. Samples were analyzed in triplicate and normalized to total protein that was determined using the Bradford Protein Assay Kit (Thermo Scientific, Rockford, IL). Inter-assay coefficient of variations (CVs) were 8.1, 6.6, and 13.6, and intra-assay CVs were 1.9, 2.3, and 5.5 for whole blood SOD, GPx, and CAT activities, respectively; inter- and intra-assay CVs for muscle SOD and GPx activities were 8.3 and 6.1% and 8.3 and 4.9%, respectively.

Malondialdehyde concentrations were quantified from sodium heparin plasma aliquots through spectral scanning spectrophotometry using a commercially available kit (Northwest Life Science Specialties, Vancouver, WA). MDA concentrations were determined at an absorbance of 532 nm and were based on the reaction of MDA with thiobarbituric acid. Samples were analyzed in triplicate with an inter-assay CV of 0.7% and an intra-assay CV of 2.7%.

Hydrogen peroxide production and concentration were determined through spectrofluorometry using freshly processed serum samples. Samples were analyzed in triplicate using an Amplex Red kit (Amplex™ Red Hydrogen Peroxide/Peroxidase Assay Kit; Invitrogen, Waltham, MA). Hydrogen peroxide production was read every 5 min and calculated over the course of 1 h. Due to the linearity of H_2_O_2_ produced over time, H_2_O_2_ concentration was determined at the endpoint read and calculated using a standard curve.

### Cytokine concentrations

Sodium heparin plasma aliquots were used to quantify cytokine concentrations using custom U-PLEX kits (Meso Scale Diagnostics, Rockville, MD) and equine “do-it-yourself” ELISA kits. Interleukin (IL)-8 (KingFisher Biotech, Inc., St Paul, MN), IL-10, and tumor necrosis factor α (TNFα; both R&D Systems, Minneapolis, MN) were quantified in the same well utilizing a specific linker to couple the biotinylated antibodies of interest. Due to concentrations outside of the range of detectability in the multi-plex format, IL-4 and IL-6 cytokines (R&D Systems) were quantified individually blocked with 1X PBS 4% BSA without specific linkers. The detection antibodies used were electrochemiluminescent conjugated. Sodium heparin plasma samples were diluted 1:4 using 1X PBS, 1% BSA, 0.05% Tween-20 and analyzed in triplicate on a MESO QuickPlex SQ 120 (Meso Scale Diagnostics, Rockville, MD, USA).

### Real time quantitative polymerase chain reaction

Gene expression of nuclear factor erythroid 2-related factor 2 (*NFE2L2; Nrf2*), heme oxygenase 1 (*HMOX1*), and *IL-1β* in whole blood from samples collected in Tempus tubes were analyzed by real -time quantitative polymerase chain reaction (RT-qPCR). RNA was isolated from whole blood as outlined by the Tempus Spin RNA Isolation Kit protocol (Applied Biosystems) and quantified using a NanoDrop (ND-1000 Spectrophotometer). Complementary DNA and qPCR were performed as published in Halloran et al. (2021). Briefly, after isolation, approximately 150 ng of RNA was combined with oligo deoxythymidine primers and SuperScript II Reverse Transcriptase (Invitrogen) to obtain first-strand cDNAs. A negative control for each treatment group and collection period that was void of reverse transcriptase was included. Quantitative polymerase chain reaction was performed with Power SYBR Green PCR Master Mix (Applied Biosystems) using the ABI Prism 7900HT system (Applied Biosystems). Sample wells included 10% diluted cDNA, 10% primer, 30% nuclease-free water, and 50% SYBR Green reaction mix in a 10-μL reaction volume. Samples were run in triplicate under the following conditions: 50°C for 2 min, 95°C for 10 min, followed by 40 cycles of 95°C for 15 s and 60°C for 1 min. All reactions were performed at an annealing temperature of 60°C. Primer sequences (Sigma-Aldrich, St Louis, MO; Table 2) were tested by using a standard curve of pooled cDNA and nuclease-free water ranging from 1:2 to 1:256 prior to use. A primer efficiency of 95–105% was deemed acceptable for use. Quantitative real-time PCR produced a cycle threshold (Ct) value for each sample. Reference genes, *RPL32* and *GUSB*, were used to normalize expression of mRNAs of interest. The resulting ΔCt was calculated by subtracting Ct_geomean of RPL32 and GUSB_ from Ct_gene of interest_. In figures, gene expression is represented as 40 − ΔCt, in which 40 is the total number of cycles run.

**Table 2.** Primer sequences used in RT-qPCR.

Gene abbreviation	Gene name	Forward/reverse primers (5′→3′)	Product size, bp	GenBank accession number
**NFE2L2/Nrf2**	Nuclear factor erythroid 2-related factor 2	F: CCCAGCAGGACATGGATTTGA R: TGCTGCAGTCGTTGAGTGAA	97	XM_023622057.1
**IL-1β**	Interleukin 1β	F: TATGTGTGTGATGCAGCTGTG R: GCTCATGCAGAACACCACTTGT	147	NM_001082526.1
**HMOX1**	Heme oxygenase-1	F: CCTGCTCAACATTCAGCTGTT R: CTCTGAGGGCGTAGGGTCTT	133	XM_023631135.1
**RPL32**	Ribosomal protein L32	F: GGGAGCAATAAGAAAACGAAGC R: CTTGGAGGAGACATTGTGAGC	138	XM_001492042.6
**GUSB**	β-Glucuronidase	F: AAGAATATGTGGTTGGAGAGCTCATCT R: CGCAAAAGGAATGCTGCACCT	136	XM_023655543.1

### Statistical analysis

Data were analyzed using linear models in SAS v9.4 with repeated measures. Sex, time, treatment, and the time × treatment interaction were included as fixed effects. Time was a repeated effect with horse (treatment) as the subject. Day 0 was included in all models as a covariate if treatment groups differed at day 0. Outliers were removed when outside of two standard deviations from the mean. All data are expressed as least square means ± SEM. Significance was declared at *P *≤ 0.05 and trends were declared when 0.05 < *P *≤ 0.10.

## Results

**Figure 1.**
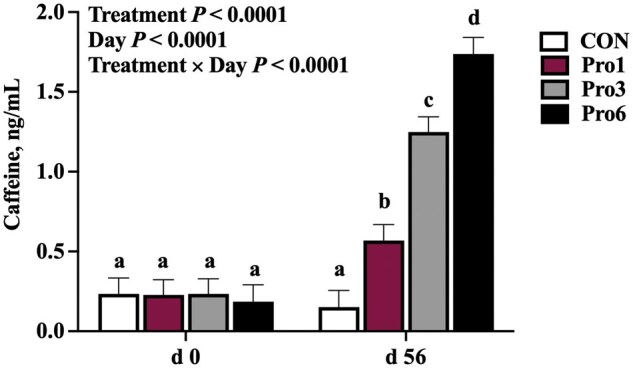
Plasma caffeine concentrations (least square means ± SEM) at day 0 and 56 of mature horses receiving 0 mg LifeVantage Protandim Nrf2 Synergizer (CON; *n* = 10); 675 mg Protandim/d (Pro1; *n* = 10); 2,025 mg Protandim/d (Pro3; *n* = 10); or 4,050 mg Protandim/d (Pro6; *n* = 10). ^a, b, c, d^Bars with different letters differ (*P *< 0.05).

Plasma caffeine concentrations were impacted by the interaction of treatment and day (*P *< 0.0001; Figure 1). Plasma caffeine did not differ amongst horses at day 0, then increased in Pro1 (*P *= 0.02), Pro3 (*P *< 0.0001), and Pro6 (*P < *0.0001) horses from day 0 to 56 but did not change in CON horses (Figure 1). A dose response in plasma caffeine was present by day 56, whereby Pro6 horses had the greatest concentration of caffeine (*P≤*0.009), followed by Pro3 horses (*P≤*0.0005), then Pro1 horses (*P≤*0.007), with CON horses having the lowest plasma caffeine concentrations (*P≤*0.007; Figure 1).

**Figure 2.**
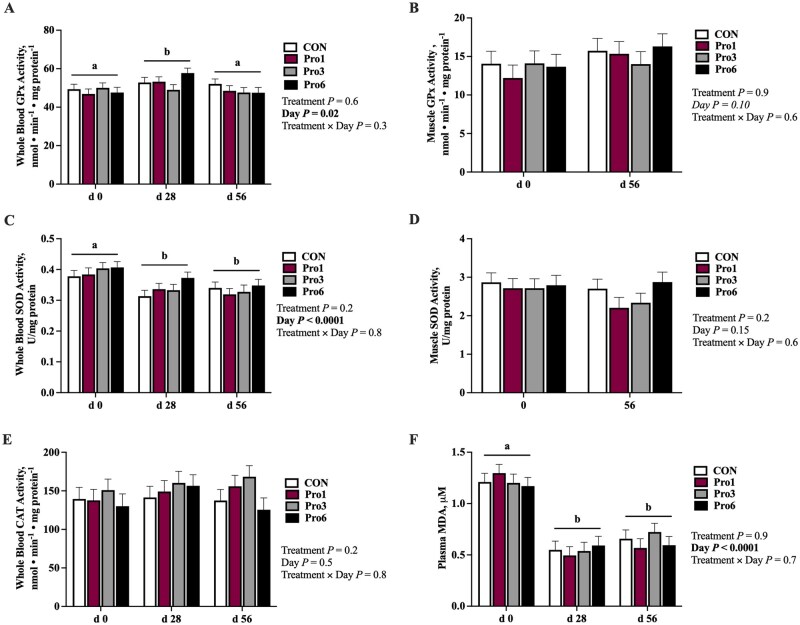
Whole blood and skeletal muscle glutathione peroxidase (GPx; A and B, respectively), superoxide dismutase (SOD; C and D, respectively) activities, whole blood catalase (CAT) activity (E), and plasma malondiadehyde (MDA) concentrations (F) at day 0, 28, and 56 of mature horses receiving 0 mg LifeVantage Protandim Nrf2 Synergizer (CON; *n* = 10); 675 mg Protandim/d (Pro1; *n* = 10); 2,025 mg Protandim/d (Pro3; *n* = 10); or 4,050 mg Protandim/d (Pro6; *n* = 10). ^a, b^Across treatments, days with different letters differ (*P *≤ 0.05). All data are presented as least square means ± SEM.

Whole blood GPx (*P *= 0.02) and SOD (*P *< 0.0001) activities and plasma MDA concentrations (*P *< 0.0001) were, and muscle GPx activity tended to be (*P *= 0.10), impacted by day but none differed by sex, treatment, or the treatment × day interaction (Figure 2). Regardless of treatment, whole blood GPx activity increased from day 0 to 28 (*P *= 0.01) and decreased from day 28 to 56 (*P *= 0.02) to be similar to day 0 at day 56 (Figure 2A). Skeletal muscle GPx activity tended to increase from day 0 to 56 regardless of treatment group (*P *= 0.10; Figure 2B). Conversely, whole blood SOD activity (Figure 2C) and plasma MDA concentration (Figure 2F) decreased from day 0 to 28 (*P *< 0.0001) and remained suppressed at day 56 (*P *< 0.0001). Skeletal muscle SOD activity (Figure 2D) and whole blood CAT activity (Figure 2E) were not impacted by supplementation, time, or sex.

**Figure 3.**
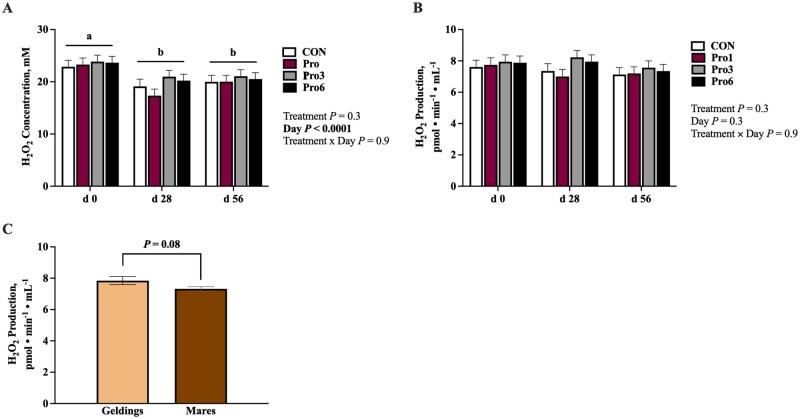
Serum hydrogen peroxide (A) concentration and (B) production at day 0, 28, and 56 of mature horses receiving 0 mg LifeVantage Protandim Nrf2 Synergizer (CON; *n* = 10); 675 mg Protandim/d (Pro1; *n* = 10); 2,025 mg Protandim/d (Pro3; *n* = 10); or 4,050 mg Protandim/d (Pro6; *n* = 10). ^a, b^Across treatments, days with different letters differ (*P *≤ 0.05). (C) Pooled serum hydrogen peroxide production of geldings (*n* = 8) and mares (*n* = 32). All data are presented as least square means ± SEM.

Serum H_2_O_2_ concentration showed only a main effect of day (*P *< 0.0001), decreasing from day 0 to 28 (*P *< 0.0001) and remaining suppressed at day 56 (*P *= 0.0007), but was not impacted by sex or dietary treatment (Figure 3A). Serum H_2_O_2_ production did not differ by treatment or time (Figure 3B) but tended to be greater in geldings than mares (*P *= 0.08; Figure 3C).

**Figure 4.**
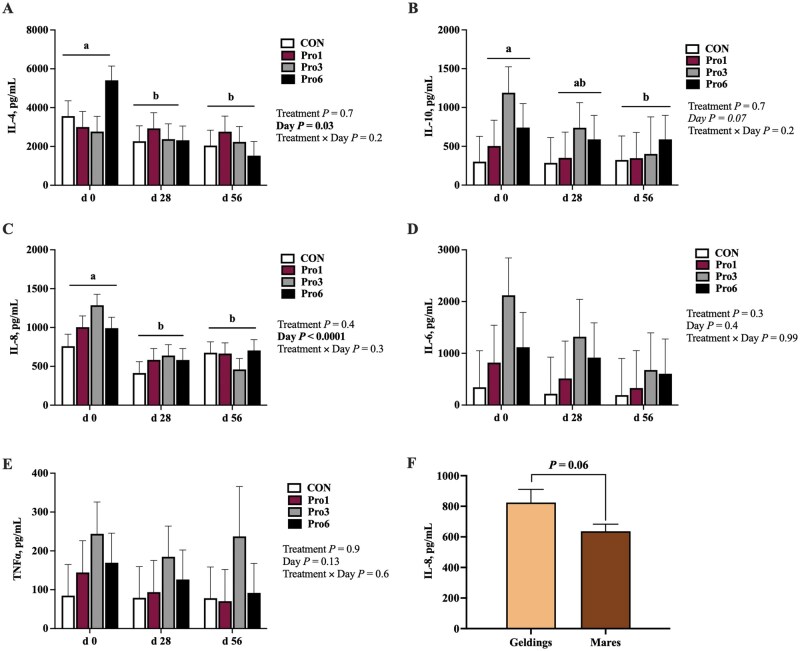
Plasma cytokine concentrations of interleukin (IL)-4 (A), IL-10 (B), IL-8 (C), IL-6 (D), and tumor necrosis factor (TNF)α (E) at day 0, 28, and 56 of mature horses receiving 0 mg LifeVantage Protandim Nrf2 Synergizer (CON; *n* = 10); 675 mg Protandim/d (Pro1; *n* = 10); 2,025 mg Protandim/d (Pro3; *n* = 10); or 4,050 mg Protandim/d (Pro6; *n* = 10). ^a, b^Across treatments, days with different letters differ (*P *≤ 0.05). (F) Pooled plasma cytokine concentrations of IL -8 of geldings (*n* = 8) and mares (*n* = 32). All data are presented as least square means ± SEM.

Plasma concentrations of IL-4 (*P *= 0.03) and IL-8 (*P < *0.0001) were, and IL-10 tended to be (*P *= 0.07), impacted by time but were unaffected by dietary treatment and the interaction of time and treatment (Figure 4). Plasma IL-4 (Figure 4A) and IL-8 (Figure 4C) concentrations decreased from day 0 to 28 (IL-4: *P *= 0.05; IL-8: *P *< 0.0001) and remained suppressed at day 56 (IL-4: *P *= 0.01; IL-8: *P *= 0.0002), while IL-10 decreased from day 0 to 56 (*P *= 0.03; Figure 4B). Plasma concentrations of both IL-6 and TNFα were unaffected by time, treatment, and the time × treatment interaction (Figure 4D and E). Geldings tended to have greater IL-8 concentrations than mares (*P *= 0.06; Figure 4F) but no other cytokine differed by sex.

**Figure 5.**
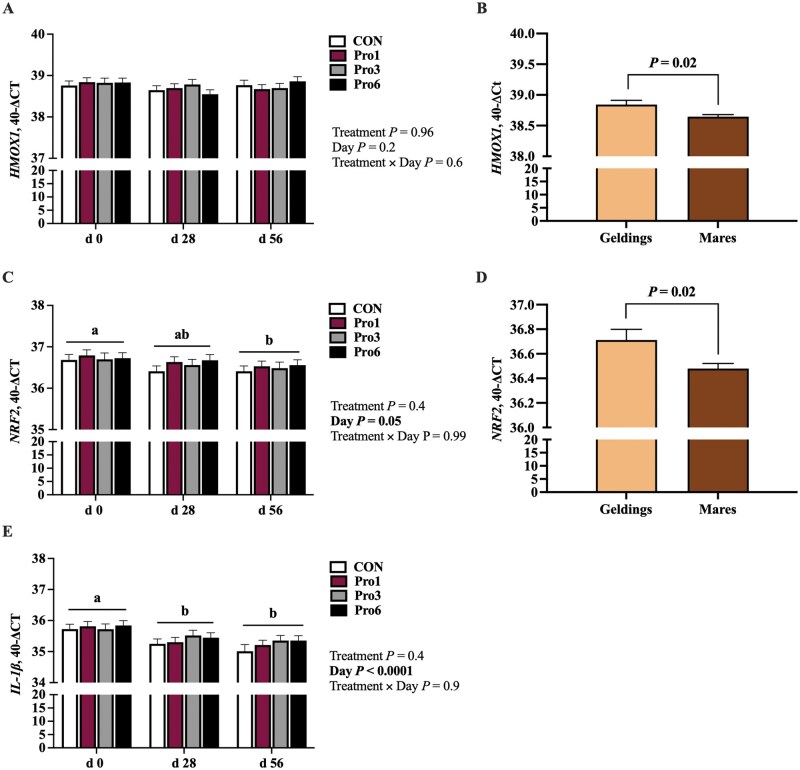
mRNA expression of *HMOX1* (A), *Nrf2* (C), and *IL-1β* (E) at day 0, 28, and 56 of mature horses receiving 0 mg LifeVantage Protandim Nrf2 Synergizer (CON; *n* = 10); 675 mg Protandim/d (Pro1; *n* = 10); 2,025 mg Protandim/d (Pro3; *n* = 10); or 4,050 mg Protandim/d (Pro6; *n* = 10). ^a, b^ Across time, treatments with different letters differ (*P *< 0.05). Pooled mRNA expression of *HMOX1* (B) and *Nrf2* (D) of geldings (*n* = 8) and mares (*n* = 32). All data are presented as least square means ± SEM.

*HMOX1* expression did not differ by treatment or time (Figure 5A) but was greater in geldings than mares (*P *= 0.02; Figure 5B). *Nrf2* (*P *= 0.05; Figure 5C) and *IL-1β* (*P *< 0.0001; Figure 5E) mRNA were impacted by the main effect of day but not by treatment or the interaction of treatment and day. *Nrf2* mRNA expression tended to decrease from day 0 to 28 (*P *= 0.1) and was greater at day 0 than day 56 (*P *= 0.02; Figure 5C); *Nrf2* expression was also greater in geldings than mares (*P *= 0.02; Figure 5D). *IL-1β* expression decreased from day 0 to 28 (*P *= 0.006) and remained suppressed at day 56 (*P *< 0.0001; Figure 5E) but did not differ by sex.

While the study was not designed to investigate age as a contributing factor, given the known inflammaging phenomenon, a brief exploratory analysis of the dataset investigating differences between aged (≥ 16 yr; *n* = 21) and mature (< 16 yr; *n* = 19) horses was performed. Details of this analysis are provided as a supplement. Aged horses had greater MDA concentrations (*P *= 0.008), whole blood catalase activity (*P *= 0.007), and muscle GPx activity (*P *= 0.04) and lesser whole blood GPx (*P *= 0.06) and SOD (*P *= 0.07) activities than mature horses (Supplementary Table S1). Aged horses also had greater IL-4, IL-6, IL-10, and TNFα concentrations (*P *< 0.05) and tended to have greater IL-8 concentrations (*P *= 0.08) than mature horses (Supplementary Table S2). The study design lacks statistical power to examine any interactions with age, so these analyses were not performed.

## Discussion

LifeVantage Protandim Nrf2 Synergizer is a proprietary blend of milk thistle, bacopa, ashwagandha, green tea, and turmeric (herbal antioxidants), which, in combination, have demonstrated a synergism working through the Nrf2 pathway (in human and mouse cell lines; Velmurugan et al., 2009) to increase enzyme activities of antioxidants, SOD, GPx, and catalase, decrease markers of lipid peroxidation, and reduce the activation of pro-inflammatory cytokines (Nelson et al., 2006; Liu et al., 2009; Velmurugan et al., 2009; Ueberschlag et al., 2016). Thus, we hypothesized that horses receiving Protandim for 56 d would have lower markers of oxidative and inflammatory stress compared to non-supplemented horses. In the current study, supplementation of Protandim increased caffeine concentrations in a dose-dependent manner. However, no effects on antioxidant status or cytokines were observed in horses with dietary supplementation of up to 4,050 mg Protandim/d, a rate equivalent to that which evoked improvements in antioxidant status and inflammatory control in humans and rodents. The disagreement with previously published literature was, therefore, unexpected.

An important observation of the current study was the apparent dose-dependent increase in plasma caffeine concentrations by day 56. One tablet (675 mg) of the Protandim Nrf2 Synergizer supplement contains 1.8 mg of naturally derived caffeine. Therefore, the increase in plasma caffeine as supplementation increased confirms that horses were consuming the supplement and that it was being properly absorbed. Notably, the plasma caffeine levels (1.8 ± 0.1 ng/mL) detected even at the highest dose of Protandim (six tablets or 4,050 mg/d) still fall well below the 20 ng/mL acceptable level for racehorses in the United States (at time of publication; International Federation of Horseracing Authorities, 2024). While caffeine is also classified as a prohibited substance by the United States Equestrian Federation, no plasma concentration defining a positive test has been specified. However, behavioral data have confirmed that performance-enhancing outcomes are not noted in horses until plasma caffeine concentrations reach 2,000 ng/mL (Queiroz-Neto et al., 2001).

The primary outcomes of the study were decreases in SOD activity, *IL-1β* mRNA, and concentrations of MDA, H_2_O_2_, IL-4, IL-8, and IL-10 in circulation over time. These alterations may be explained by the decreasing environmental temperature over the course of the study period. Heat exposure has extensively been shown to increase ROS in poultry, lead to an increase in incidences of oxidative stress, and induce IL-8 transcription (Altan et al., 2003; Mujahid et al., 2005; Singh et al., 2008). The higher temperatures and greater sunlight exposure may have resulted in excess ROS production at the beginning of the study period in September, then decreased over time with the temperature and sunlight declines in October and November. Whole blood and skeletal muscle GPx activity did not follow a similar pattern, however, and the reasons for their increases at day 28 in blood and day 56 in muscle are uncertain.

Expression of *Nrf2* also decreased throughout the study period. Nuclear factor erythroid 2-related factor is a transcription factor that plays a role in the activation of antioxidant enzymes and repression of inflammation. The elevation in *Nrf2* at day 0 may have been in response to increased oxidative stress (evidenced by elevated H_2_O_2_ and MDA concentrations) that was also present at day 0 to activate enzymes like SOD. Similarly, the presence of inflammation at day 0 may have triggered Nrf2 activation, which has been shown to inhibit transcription of pro-inflammatory cytokines, namely IL-1β and IL-6 (Kobayashi et al., 2016). As a decline in inflammation and oxidative stress occurred, a concomitant decrease in Nrf2 activation likely followed. In contrast, expression of *HMOX1*, a downstream target of Nrf2 with antioxidant and anti-inflammatory roles (Loboda et al., 2016), was unchanged throughout the study.

The sex effects observed in serum H_2_O_2_ production and whole blood *Nrf2* and *HMOX1* expression are largely unexplained. In humans, estrogen has been shown to play a protective role in cardiovascular disease by improving heart function while also having antioxidant potential (Czubryt et al., 2006; Kander et al., 2017). Further studies have revealed that oxidative stress and ROS production are greater in males compared to females and females overall have a greater antioxidant defense system compared to males (Ide et al., 2002; Matarrese et al., 2011; Bhatia et al., 2012). These findings may suggest that decreased oxidative stress in combination with a primed antioxidant defense system may contribute to the lower levels of serum H_2_O_2_ produced in mares compared to geldings. However, it should be noted that no other sex differences were found amongst the antioxidant and oxidative stress markers measured, so this cannot fully explain the sex differences observed. The use of geldings (neutered males) versus intact males may be a potential explanation for the discrepancies.

Similar sex effects were observed in plasma IL-8 concentration, which was greater in geldings than mares. The greater H_2_O_2_ produced in geldings in this study could have induced IL-8 synthesis. In human bronchial epithelial cells, exposure to H_2_O_2_ induced IL-8 synthesis in a concentration-dependent manner as a result of mitogen-activated protein kinase, which is responsible for *IL-8* mRNA stability and expression (Pelaia et al., 2004; Bhattacharyya et al., 2011). Similar in vitro analysis of human colon adenocarcinoma cell line Caco-2 demonstrated increased IL-8 production in filter-grown cells 24 h post-introduction of 1 and 10 mM H_2_O_2_ (Németh et al., 2007). Importantly, mares and geldings were not equally represented in this study so sex differences should be interpreted with caution.

The reason for the lack of effect of supplementation on antioxidant, oxidative stress, and inflammatory markers in the current study is unclear. Treatment groups were balanced for age, sex, BW, and housing location (pasture) so the study was designed to account for variations due to any of these factors. Random genetic and/or physiological variations between horses may be a potential explanation for the findings, which is supported by wide standard errors of the means for most cytokines reported herein. This wide inter-animal variation in circulating cytokines has been reported in horses previously (Moellerberndt et al., 2023; Artman et al., 2024). It is also possible that some of the horses may have had underlying conditions due to normal aging dynamics that did not present clinically. For instance, IL-6, IL-8, and interferon-gamma (IFN-γ) concentrations have been shown to be elevated in horses ≥16 yr old, and older horses also had greater IL-6/IL-10 and TNFα/IL-10 ratios; this phenomenon is collectively referred to as inflammaging (McFarlane and Holbrook, 2008). From a disease perspective, TNFα, IL-6, and IL-10 have been associated with osteoarthritis, a common condition in older horses (Miller et al., 2014). In the current study, aged horses (≥ 16 yr) had altered antioxidant status and greater cytokine concentrations than mature horses (< 16 yr). The study design lacked statistical power to investigate age × treatment × time interactions but it is possible that the supplement may be more beneficial to older horses experiencing inflammaging compared to mature horses with a less inflammatory cellular environment.

Another potential explanation for the lack of impacts of dietary treatment observed could be that the horses were sedentary and in a relatively low-stress environment. Standardbreds subjected to a single bout of exhaustive exercise exhibited increased whole blood TNFα concentrations and reduced IL-1 receptor antagonist concentrations (an anti-inflammatory marker; Hale et al., 2023). Unfit Standardbred mares similarly had elevated blood *IFN-γ*, *IL-1*, and *TNFα* expression and muscle *IFN-γ*, *IL-6*, and *TNFα* expression following a high-speed treadmill test (Liburt et al., 2010). Extensive literature has demonstrated increases in oxidative stress post-exercise in horses (Mills et al., 1996; White et al., 2016; White and Warren, 2017; Nemec Svete et al., 2021). As such, Protandim’s anti-inflammatory and antioxidant benefits may be more appreciated in horses undergoing a stressful stimulus, such as exercise.

Overall, there were no impacts of Protandim supplementation on the antioxidant and inflammatory status of mature sedentary horses as measured by GPx activity, SOD activity, MDA concentration, H_2_O_2_ production and concentration, cytokine (IL-4, IL-6, IL-8, IL-10, TNFα) concentrations, and *Nrf2*, *HMOX1*, and *IL-1β* mRNA expression. There was a dose-dependent effect of supplementation on plasma caffeine concentration, as expected, but circulating caffeine remained below regulatory limits for competition horses. To determine the true implications of Protandim supplementation on the horse, future research should evaluate the impacts of an increased dosage and the introduction of oxidative and/or inflammatory insult on the horse’s antioxidant defense and inflammatory systems.

## Supplementary Material

skaf433_Supplementary_Data

## Abbreviations:

BCS: Body condition score
BW: Body weight
CAT: Catalase
DAMP: Damage-associated molecular pattern
DM: Dry matter
GPx: Glutathione peroxidase
H_2_O_2_: Hydrogen peroxide
HMOX1: Heme oxygenase-1
IFN-γ: Interferon-gamma
IL: Interleukin
MDA: Malondialdehyde
NF-κB: nuclear factor-kappa B
NLRP3: NOD-like receptor family pyrin domain containing 3
Nrf2/NFE2L2: Nuclear factor erythroid 2-related factor
RT-qPCR: Real -time quantitative polymerase chain reaction
ROS: Reactive oxygen species
SOD: Superoxide dismutase
TLR: Toll-like receptor
TNFα: Tumor necrosis factor alpha

## References

[skaf433-B1] Adams A. , Breathnach C., Katepalli M., Kohler K., Horohov D. 2008. Advanced age in horses affects divisional history of T cells and inflammatory cytokine production. Mech. Ageing Dev. 129(11):656–664. 18926847 10.1016/j.mad.2008.09.004

[skaf433-B2] Adams A. A. , Katepalli M. P., Kohler K., Reedy S. E., Stilz J., Vick M. M., Fitzgerald B. P., Lawrence L. M., Horohov D. W. 2009. Effect of body condition, body weight and adiposity on inflammatory cytokine responses in old horses. Vet. Immunol. Immunopathol. 127(3–4):286–294. 19097648 10.1016/j.vetimm.2008.10.323

[skaf433-B3] Artman J. L. , Wesolowski L. T., Semanchik P. L., Isles J. K., Norton S. A., White-Springer S. H. 2024. Local and systemic responses to repeated gluteal muscle microbiopsies in mature sedentary horses. J Equine Vet Sci. 136:105070. 10.1016/j.jevs.2024.105070 38642813

[skaf433-B4] Altan Ö. , Pabuçcuoğlu A., Altan A., Konyalioğlu S., Bayraktar H. 2003. Effect of heat stress on oxidative stress, lipid peroxidation and some stress parameters in broilers. Br. Poult. Sci. 44(4):545–550. 10.1080/00071660310001618334 14584844

[skaf433-B5] Bechtel P , Kline K. 1987. Muscle fiber type changes in the middle gluteal of quarter and standardbred horses from birth through one year of age. Proc. Int. Conf. Equine Exer. Phys. No. 2. p. 265–270. Davis, Calif.: ICEEP Publications, 1987, San Diego, CA.

[skaf433-B6] Bhatia K. , Elmarakby A. A., EL -Remessy A. B., Sullivan J. C. 2012. Oxidative stress contributes to sex differences in angiotensin II-mediated hypertension in spontaneously hypertensive rats. Am. J. Physiol. Regul. Integr. Comp. Physiol. 302(2):R274–R282. 10.1152/ajpregu.00546.2011 22049231 PMC3349386

[skaf433-B7] Bhattacharyya S. , Gutti U., Mercado J., Moore C., Pollard H. B., Biswas R. 2011. MAPK signaling pathways regulate IL -8 mRNA stability and IL -8 protein expression in cystic fibrosis lung epithelial cell lines. Am. J. Physiol. Lung Cell. Mol. Physiol. 300(1):L81–L87. 10.1152/ajplung.00051.2010 20952496 PMC3023294

[skaf433-B8] Bruns D. R. , Ehrlicher S. E., Khademi S., Biela L. M., Peelor F. F., Miller B. F., Hamilton K. L. 2018. Differential effects of vitamin C or protandim on skeletal muscle adaptation to exercise. J. Appl. Physiol. (1985). 125(2):661–671. 10.1152/japplphysiol.00277.2018 29856263 PMC6139515

[skaf433-B10] Czubryt M. P. , Espira L., Lamoureux L., Abrenica B. 2006. The role of sex in cardiac function and disease. Can. J. Physiol. Pharmacol. 84(1):93–109. 10.1139/y05-151 16845894

[skaf433-B11] DeNotta S , McFarlane D. 2023. Immunosenescence and inflammaging in the aged horse. Immun. Ageing. 20(1):2. 10.1186/s12979-022-00325-5 36609345 PMC9817422

[skaf433-B12] Dominic A. , Le N. T., Takahashi M. 2022. Loop between NLRP3 inflammasome and reactive oxygen species. Antioxid. Redox Signal. 36(10–12):784–796. 10.1089/ars.2020.8257 34538111

[skaf433-B13] Ďuračková Z. 2010. Some current insights into oxidative stress. Physiol. Res. 59(4):459–469. 10.33549/physiolres.931844 19929132

[skaf433-B14] Hale J. N. , Hughes K. J., Hall S., Labens R. 2023. The effect of exercise on cytokine concentration in equine autologous conditioned serum. Equine Vet. J. 55(3):551–556. 10.1111/evj.13586 35569120

[skaf433-B15] Henneke D. R. , Potter G. D., Kreider J. L., Yeates B. F. 1983. Relationship between condition score, physical measurements and body fat percentage in mares. Equine. Vet. J. 15(4):371–372. 10.1111/j.2042-3306.1983.tb01826.x 6641685

[skaf433-B16] Halloran K. M. , Hoskins E. C., Stenhouse C., Moses R. M., Dunlap K. A., Satterfield M. C., Seo H., Johnson G. A., Wu G., Bazer F. W. 2021. Pre-implantation exogenous progesterone and pregnancy in sheep. II. Effects on fetaL -placental development and nutrient transporters in late pregnancy. J. Anim. Sci. Biotechnol. 12(1):46–65. 10.1186/s40104-021-00567-1 33827696 PMC8028684

[skaf433-B17] Horohov D. W. , Sinatra S. T., Chopra R. K., Jankowitz S., Betancourt A., Bloomer R. J. 2012. The effect of exercise and nutritional supplementation on proinflammatory cytokine expression in young racehorses during training. J Equine Vet Sci. 32(12):805–815. 10.1016/j.jevs.2012.03.017

[skaf433-B18] Hybertson B. M. , Gao B., Bose S. K., McCord J. M. 2011. Oxidative stress in health and disease: the therapeutic potential of Nrf2 activation. Mol. Aspects Med. 32(4–6):234–246. 10.1016/j.mam.2011.10.006 22020111

[skaf433-B19] Ide T. , Tsutsui H., Ohashi N., Hayashidani S., Suematsu N., Tsuchihashi M., Tamai H., Takeshita A. 2002. Greater oxidative stress in healthy young men compared with premenopausal women. Arterioscler Thromb. Vasc. Biol. 22(3):438–442. 10.1161/hq0302.104515 11884287

[skaf433-B20] International Federation of Horseracing Authorities. 2024. Residue limits—urine and plasma. https://www.ifhaonline.org/default.asp? section=IABRW&area=18.

[skaf433-B21] Jakubczyk K. , Drużga A., Janda K., Skonieczna-Żydecka K. 2020. Antioxidant potential of curcumin—a meta-analysis of randomized clinical trials. Antioxidants. 9(11):1092–1104. 10.3390/antiox9111092 33172016 PMC7694612

[skaf433-B22] Kander M. C. , Cui Y., Liu Z. 2017. Gender difference in oxidative stress: a new look at the mechanisms for cardiovascular diseases. J. Cell. Mol. Med. 21(5):1024–1032. 10.1111/jcmm.13038 27957792 PMC5387169

[skaf433-B23] Kędzierski W. , Janczarek I., Kowalik S., Jamioł M., Wawak T., Borsuk G., Przetacznik M. 2020. Bee pollen supplementation to aged horses influences several blood parameters. J. Equine Vet. Sci. 90:103024. 10.1016/j.jevs.2020.103024 32534787

[skaf433-B24] Kim J. S. , Lee Y. H., Chang Y. U., Yi H. K. 2017. PPARγ regulates inflammatory reaction by inhibiting the MAPK/NF-κB pathway in C2C12 skeletal muscle cells. J. Physiol. Biochem. 73(1):49–57. 10.1007/s13105-016-0523-3 27718123

[skaf433-B25] Kobayashi E. H. , Suzuki T., Funayama R., Nagashima T., Hayashi M., Sekine H., Tanaka N., Moriguchi T., Motohashi H., Nakayama K. et al. 2016. Nrf2 suppresses macrophage inflammatory response by blocking proinflammatory cytokine transcription. Nat. Commun. 7:11624. 10.1038/ncomms11624 27211851 PMC4879264

[skaf433-B26] Koenig A , Buskiewicz-Koenig I. A. 2022. Redox activation of mitochondrial DAMPs and the metabolic consequences for development of autoimmunity. Antioxid. Redox Signal. 36(7–9):441–461. 10.1089/ars.2021.0073 35352943 PMC8982130

[skaf433-B27] Konopka A. R. , Laurin J. L., Musci R. V., Wolff C. A., Reid J. J., Biela L. M., Zhang Q., Peelor F. F., Melby C. L., Hamilton K. L. et al. 2017. Influence of Nrf2 activators on subcellular skeletal muscle protein and DNA synthesis rates after 6 weeks of milk protein feeding in older adults. Geroscience. 39(2):175–186. 10.1007/s11357-017-9968-8 28283797 PMC5411371

[skaf433-B28] Liburt N. R. , Adams A. A., Betancourt A., Horohov D. W., McKeever K. H. 2010. Exercise-induced increases in inflammatory cytokines in muscle and blood of horses. Equine Vet. J. 42(38):280–288. 10.1111/j.2042-3306.2010.00275.x 21059019

[skaf433-B29] Liu J. , Gu X., Robbins D., Li G., Shi R., McCord J. M., Zhao Y. 2009. Protandim, a fundamentally new antioxidant approach in chemoprevention using mouse two-stage skin carcinogenesis as a model. PLoS One. 4(4):e5284. 10.1371/journal.pone.0005284 19384424 PMC2668769

[skaf433-B30] Loboda A. , Damulewicz M., Pyza E., Jozkowicz A., Dulak J. 2016. Role of Nrf2/HO-1 system in development, oxidative stress response and diseases: an evolutionarily conserved mechanism. Cell. Mol. Life Sci. 73(17):3221–3247. 10.1007/s00018-016-2223-0 27100828 PMC4967105

[skaf433-B31] Matarrese P. , Colasanti T., Ascione B., Margutti P., Franconi F., Alessandri C., Conti F., Riccieri V., Rosano G., Ortona E. et al. 2011. Gender disparity in susceptibility to oxidative stress and apoptosis induced by autoantibodies specific to RLIP76 in vascular cells. Antioxid. Redox Signal. 15(11):2825–2836. 10.1089/ars.2011.3942 21671802

[skaf433-B32] McFarlane D , Holbrook T. C. 2008. Cytokine dysregulation in aged horses and horses with pituitary pars intermedia dysfunction. J. Vet. Intern. Med. 22(2):436–442. 10.1111/j.1939-1676.2008.0076.x 18371032

[skaf433-B33] Miller A. B. , Harris P. A., Barker V. D., Adams A. A. 2021. Short-term transport stress and supplementation alter immune function in aged horses. PLoS One. 16(8):e0254139. 10.1371/journal.pone.0254139 34411137 PMC8376036

[skaf433-B34] Miller R. E. , Miller R. J., Malfait A.-M. 2014. Osteoarthritis joint pain: the cytokine connection. Cytokine. 70(2):185–193. 10.1016/j.cyto.2014.06.019 25066335 PMC4254338

[skaf433-B35] Mills P. C. , Smith N. C., Casas I., Harris P., Harris R. C., Marlin D. J. 1996. Effects of exercise intensity and environmental stress on indices of oxidative stress and iron homeostasis during exercise in the horse. Eur. J. Appl. Physiol. Occup. Physiol. 74(1–2):60–66. 10.1007/bf00376495 8891501

[skaf433-B36] Moellerberndt J. , Hagen A., Niebert S., Büttner K., Burk J. 2023. Cytokines in equine platelet lysate and related blood products. Front. Vet. Sci. 10:1117829. 10.3389/fvets.2023.1117829 36968472 PMC10033973

[skaf433-B37] Mujahid A. , Yoshiki Y., Akiba Y., Toyomizu M. 2005. Superoxide radical production in chicken skeletal muscle induced by acute heat stress. Poult. Sci. 84(2):307–314. 10.1093/ps/84.2.307 15742968

[skaf433-B38] National Research Council. 2007. Nutrient requirements of horses. 6th ed. Washington, DC: National Academies Press.

[skaf433-B39] Nelson S. K. , Bose S. K., Grunwald G. K., Myhill P., McCord J. M. 2006. The induction of human superoxide dismutase and catalase in vivo: a fundamentally new approach to antioxidant therapy. Free Radic. Biol. Med. 40(2):341–347. 10.1016/j.freeradbiomed.2005.08.043 16413416

[skaf433-B40] Nemec Svete A. , Vovk T., Bohar Topolovec M., Kruljc P. 2021. Effects of vitamin E and coenzyme Q10 supplementation on oxidative stress parameters in untrained leisure horses subjected to acute moderate exercise. Antioxidants (Basel). 10(6):908–920. 10.3390/antiox10060908 34205129 PMC8227526

[skaf433-B41] Németh E. , Halász A., Baráth A., Domokos M., Gálfi P. 2007. Effect of hydrogen peroxide on interleukin-8 synthesis and death of Caco-2 cells. Immunopharmacol. Immunotoxicol. 29(2):297–310. 10.1080/08923970701513443 17849273

[skaf433-B42] O’Rourke S. A. , Shanley L. C., Dunne A. 2024. The Nrf2-HO-1 system and inflammaging. Front. Immunol. 15:1457010. 10.3389/fimmu.2024.1457010 39380993 PMC11458407

[skaf433-B43] Osada K. , Takahashi M., Hoshina S., Nakamura M., Nakamura S., Sugano M. 2001. Tea catechins inhibit cholesterol oxidation accompanying oxidation of low density lipoprotein in vitro. Comp. Biochem. Physiol. C Toxicol. Pharmacol. 128(2):153–164. 10.1016/s1532-0456(00)00192-7 11239828

[skaf433-B44] Owen R. N. , Semanchik P. L., Latham C. M., Brennan K. M., White S. 2022. Elevated dietary selenium rescues mitochondrial capacity impairment induced by decreased vitamin E intake in young exercising horses. J. Anim. Sci. 100(8):skac172. 10.1093/jas/skac172 35908793 PMC9339289

[skaf433-B45] Pelaia G. , Cuda G., Vatrella A., Gallelli L., Fratto D., Gioffrè V., D’Agostino B., Caputi M., Maselli R., Rossi F. et al. 2004. Effects of hydrogen peroxide on MAPK activation, IL -8 production and cell viability in primary cultures of human bronchial epithelial cells. J. Cell. Biochem. 93(1):142–152. 10.1002/jcb.20124 15352171

[skaf433-B46] Queiroz-Neto A. , Zamur G., Carregaro A., Mataqueiro M., Salvadori M., Azevedo C., Harkins J., Tobin T. 2001. Effects of caffeine on locomotor activity of horses: determination of the no-effect threshold. J. Appl. Toxicol. 21(3):229–234. 10.1002/jat.748 11404835

[skaf433-B47] Qureshi M. M. , McClure W. C., Arevalo N. L., Rabon R. E., Mohr B., Bose S. K., McCord J. M., Tseng B. S. 2010. The dietary supplement protandim decreases plasma osteopontin and improves markers of oxidative stress in muscular dystrophy mdx mice. J. Diet. Suppl. 7(2):159–178. 10.3109/19390211.2010.482041 20740052 PMC2926985

[skaf433-B48] Sies H , Jones D. P. 2020. Reactive oxygen species (ROS) as pleiotropic physiological signalling agents. Nat. Rev. Mol. Cell Biol. 21(7):363–383. 10.1038/s41580-020-0230-3 32231263

[skaf433-B49] Singh I. S. , Gupta A., Nagarsekar A., Cooper Z., Manka C., Hester L., Benjamin I. J., He J., Hasday J. D. 2008. Heat shock co-activates interleukin-8 transcription. Am. J. Respir. Cell. Mol. Biol. 39(2):235–242. 10.1165/rcmb.2007-0294OC 18367728 PMC2542457

[skaf433-B51] Ueberschlag S. L. , Seay J. R., Roberts A. H., DeSpirito P. C., Stith J. M., Folz R. J., Carter K. A., Weiss E. P., Zavorsky G. S. 2016. The effect of Protandim^®^ supplementation on athletic performance and oxidative blood markers in runners. PLoS One. 11(8):e0160559. 10.1371/journal.pone.0160559 27513339 PMC4981460

[skaf433-B52] Velmurugan K. , Alam J., McCord J. M., Pugazhenthi S. 2009. Synergistic induction of heme oxygenase-1 by the components of the antioxidant supplement protandim. Free Radic. Biol. Med. 46(3):430–440. 10.1016/j.freeradbiomed.2008.10.050 19056485

[skaf433-B53] Vomund S. , Schäfer A., Parnham M. J., Brüne B., and Von Knethen A. 2017. Nrf2, the master regulator of anti-oxidative responses. Int. J. Mol. Sci. 18(12):2772–2783. 10.3390/ijms18122772 29261130 PMC5751370

[skaf433-B54] White S. H. , Johnson S. E., Bobel J. M., Warren L. K. 2016. Dietary selenium and prolonged exercise alter gene expression and activity of antioxidant enzymes in equine skeletal muscle. J. Anim. Sci. 94(7):2867–2878. 10.2527/jas.2016-0348 27482673

[skaf433-B55] White S. H. , Warren L. K. 2017. Submaximal exercise training, more than dietary selenium supplementation, improves antioxidant status and ameliorates exercise-induced oxidative damage to skeletal muscle in young equine athletes. J. Anim. Sci. 95(2):657–670. 10.2527/jas.2016.1130 29432539

