# Supplementary material for: Limited impacts of dietary Protandim Nrf2 Synergizer on antioxidant and inflammatory status of mature, sedentary horses
Source: J Anim Sci. 2025 Dec 15;104:skaf433. doi: 10.1093/jas/skaf433 (PMC12918311; doi:10.1093/jas/skaf433)
Supplement: skaf433_Supplementary_Data [file skaf433_supplementary_data.zip › Supplement.docx]

Horses were grouped by age, where mature were horses < 16 years of age and aged were horses $\geq$ 16 years of age.

***Statistical Analysis – Supplemental Tables***

Data were analyzed using linear models in SAS v9.4 with repeated measures. Age was included as a fixed effect and time was a repeated effect with horse(treatment) as the subject. Outliers were removed when outside of two standard deviations from the mean. All data are expressed as least square means ± SEM. Significance was declared at *P* ≤ 0.05 and trends were declared when 0.05 < *P* ≤ 0.10.
