# Supplementary material for: Limited impacts of dietary Protandim Nrf2 Synergizer on antioxidant and inflammatory status of mature, sedentary horses
Source: J Anim Sci. 2025 Dec 15;104:skaf433. doi: 10.1093/jas/skaf433 (PMC12918311; doi:10.1093/jas/skaf433)
Supplement: skaf433_Supplementary_Data [file skaf433_supplementary_data.zip › Supplemental Tables.docx]

**Supplemental Table 1.** Measures of oxidative stress and antioxidant status (mean $\pm$ SEM) of mature (< 15 years; n = 19) and aged ($\geq$16 years; n = 21) horses receiving up to 4,050 mg/d LifeVantage Protandim Nrf2 Synergizer for 56 d.

|  | **Mature** | **Aged** |  | ***P*-value** |
| --- | --- | --- | --- | --- |
| ***Serum*** |  |  |  |  |
| H_2_O_2_ concentration, mM | 20.48 $\pm$ 0.90 | 20.89 $\pm$ 0.84 |  | 0.746 |
| H_2_O_2_ production,  pmol • min^-1^ • mL^-1^ | 7.52 $\pm$ 0.27 | 7.48 $\pm$ 0.25 |  | 0.918 |
| ***Plasma*** |  |  |  |  |
| Malondialdehyde, μM | 0.67 $\pm$ 0.07 | 0.94 $\pm$ 0.07 |  | **0.008** |
| ***Whole Blood*** |  |  |  |  |
| Glutathione peroxidase, nmol • min^-1^ • mg protein^-1^ | 52.32 $\pm$ 1.15 | 49.18 $\pm$ 1.12 |  | **0.057** |
| Superoxide dismutase, U/mg protein | 0.372 $\pm$ 0.010 | 0.345 $\pm$ 0.010 |  | *0.074* |
| Catalase,  nmol • min^-1^ • mg protein^-1^ | 131.92 $\pm$ 6.48 | 157.33 $\pm$ 6.21 |  | **0.007** |
| ***Muscle*** |  |  |  |  |
| Glutathione peroxidase, nmol • min^-1^ • mg protein^-1^ | 12.57 $\pm$ 0.84 | 15.06 $\pm$ 0.79 |  | **0.036** |
| Superoxide dismutase, U/mg protein | 2.61 $\pm$ 0.13 | 2.81 $\pm$ 0.12 |  | 0.257 |

**Supplemental Table 2.** Whole blood *Interleukin (IL)-1β* expression and plasma IL-4, IL-6, IL-10, and TNFα concentrations (mean $\pm$ SEM) of mature (< 15 years; n = 19) and aged ($\geq$16 years; n = 21) horses receiving up to 4,050 mg/d LifeVantage Protandim Nrf2 Synergizer for 56 d.

|  | **Mature** | **Aged** |  | ***P*-value** |
| --- | --- | --- | --- | --- |
| ***Whole Blood*** |  |  |  |  |
| *IL-1β*, 40 - dCt | 35.52 $\pm$ 0.09 | 35.44 $\pm$ 0.07 |  | 0.474 |
| ***Plasma*** |  |  |  |  |
| IL-4, pg/mL | 1559 $\pm$ 647 | 4069 $\pm$ 640 |  | **0.009** |
| IL-6, pg/mL | 318 $\pm$ 334 | 1396 $\pm$ 341 |  | **0.030** |
| IL-8, pg/mL | 587 $\pm$ 74 | 768 $\pm$ 70 |  | *0.083* |
| IL-10, pg/mL | 242 $\pm$ 184 | 835 $\pm$ 188 |  | **0.031** |
| TNFα, pg/mL | 51 $\pm$ 52 | 222 $\pm$ 52 |  | **0.025** |

**Supplemental Table 3.** Whole blood *NFE2L2* and *HMOX1* expression (mean $\pm$ SEM) of mature (< 15 years; n = 19) and aged ($\geq$16 years; n = 21) horses receiving up to 4,050 mg/d LifeVantage Protandim Nrf2 Synergizer for 56 d.

|  | **Mature** | **Aged** |  | ***P*-value** |
| --- | --- | --- | --- | --- |
| *Nrf2*, 40 - dCt | 36.46 $\pm$ 0.07 | 36.55 $\pm$ 0.05 |  | 0.298 |
| *HMOX1*, 40 - dCt | 38.59 $\pm$ 0.08 | 38.74 $\pm$ 0.06 |  | 0.129 |
